# Rates of pneumonia among children and adults with chronic medical conditions in Germany

**DOI:** 10.1186/s12879-015-1162-y

**Published:** 2015-10-30

**Authors:** Stephen I. Pelton, Kimberly M. Shea, Raymond A. Farkouh, David R. Strutton, Sebastian Braun, Christian Jacob, Rogier Klok, Elana S. Gruen, Derek Weycker

**Affiliations:** Boston University School of Public Health, Boston, MA USA; Boston Medical Center, Boston, MA USA; Pfizer Inc., Collegeville, PA USA; Xcenda GmbH, Hannover, Germany; Pfizer bv, Capelle a/d IJssel, Netherlands; Policy Analysis Inc. (PAI), Brookline, MA USA; Maxwell Finland Laboratory for Infectious Diseases, 670 Albany Street, 6th Floor, Boston, MA 02118 USA

**Keywords:** *Streptococcus pneumoniae*, Pneumococcal infections, Pneumonia, Comorbidity, Germany

## Abstract

**Background:**

The objective of this study is to evaluate rates of all-cause pneumonia among “at-risk” and “high-risk” children and adults in Germany—in comparison with age-stratified healthy counterparts—during the period following the 2006 recommendation for universal immunization of infants with pneumococcal conjugate vaccine.

**Methods:**

Retrospective cohort design and healthcare claims information for 3.4 M persons in Germany (2009–2012) were employed. Study population was stratified by age and risk profile (healthy, “at-risk” [with chronic medical conditions], and “high-risk” [immunocompromised]). At-risk and high-risk conditions, as well as episodes of all-cause pneumonia, were identified via diagnosis, procedure, and drug codes.

**Results and discussion:**

Rates of all-cause pneumonia were 1.7 (95 % CI 1.7-1.8) to 2.5 (2.4-2.5) times higher among children and adults with at-risk conditions versus healthy counterparts, and 1.8 (1.8-1.9) to 4.1 (4.0-4.2) times higher among children and adults with high-risk conditions. Rates of all-cause pneumonia among at-risk persons increased in a graded and monotonic fashion with increasing numbers of conditions (i.e., risk stacking).

**Conclusions:**

An increased risk for all-cause pneumonia in German children and adults with a spectrum of medical conditions persists in the era of widespread pneumococcal vaccination, and pneumonia risk in persons with ≥2 at-risk conditions is comparable or higher than those with high-risk conditions.

**Electronic supplementary material:**

The online version of this article (doi:10.1186/s12879-015-1162-y) contains supplementary material, which is available to authorized users.

## Background

Community-acquired pneumonia (CAP) is a frequent cause of hospitalization and death in Germany. The incidence of CAP has been estimated to be 3.7-10.1/1000 inhabitants [1], and over 259,000 German adults were estimated to have been hospitalized for CAP in 2013 [2]. Ewig and colleagues observed that the incidence of CAP increased with advancing age, and that overall in-hospital mortality of CAP patients was 14 %, with the highest mortality in persons with comorbid conditions; among persons with malignancies, dementia, and pulmonary diseases (other than chronic obstructive pulmonary disease [COPD]), mortality was more than two-fold higher than among those with no known underlying conditions. Importantly, mortality risk was highest in the first days after admission, suggesting that prevention strategies may be more important than therapeutic approaches to reduce mortality [3].

*Streptococcus pneumoniae* (pneumococcus) has been reported to be one of the most common causes of ambulatory, hospitalized, and severe pneumonia among adults throughout Europe [4, 5]. While it is widely recognized that persons with immunocompromising conditions, as well as those with certain chronic illnesses, were at increased risk of pneumococcal disease prior to the widespread introduction of seven-valent pneumococcal conjugate vaccine (PCV7) in the US and western Europe, recent assessments of disease risk in populations following mass vaccination of children with PCVs are limited. The indirect effect of childhood immunization has led to impressive declines in the incidence of invasive pneumococcal disease in US adults [6–11], but more variable herd effects in western Europe [12]. Further confirmation from additional populations as to the magnitude of the increased risk of pneumococcal disease associated with certain chronic medical conditions following introduction of pneumococcal conjugate vaccines would be valuable for developing future vaccine policies for the prevention of pneumonia.

We therefore employed a large German research database to estimate rates of all-cause pneumonia—as a proxy for pneumococcal disease—among persons with and without one or more of the chronic illnesses included by the German Committee on Vaccination (Ständige Impfkommission [STIKO]) and/or the US Advisory Committee for Immunization Practices (ACIP) as indications for pneumococcal vaccination. We also examined disease rates among persons with other conditions that might increase infection risk based on limited data from other studies, including three autoimmune diseases—rheumatoid arthritis, systemic lupus erythematosus, and Crohn’s disease—as well as neuromuscular (chiefly cerebral palsy)/seizure disorders [13–16]. Finally, we examined the impact of risk stacking among the at-risk population by estimating disease rates within subgroups defined on the basis of the number of concurrent conditions.

## Methods

### Data design and source

A retrospective cohort design and data from the Health Risk Institute (HRI) Research Database spanning 2008–2012 were employed. The HRI Database comprises medical and drug claims from an age and gender representative sample of 3.4 million persons covered by the statutory health care system in Germany, approximately 4 % of the total population. The HRI Scientific Board approved our study and granted access to the HRI Research Database.

Data available from each medical claim include date/quarter of service, place of service, diagnoses (International Statistical Classification of Diseases and Related Health Problems, 10^th^ revision, German Modification [ICD-10-GM]), procedures performed/services rendered, and quantity of services. Data available for each drug claim include the agent dispensed (as set forth by the Anatomical Therapeutic Chemical [ATC] System), dispensing/prescription date, and quantity dispensed. Medical and drug claims also include amounts paid (i.e., reimbursed) to providers by health insurers. Selected demographic and eligibility information (including age/year of birth, sex, dates of enrollment) also is available for persons in the HRI Database. All data can be arrayed to provide a detailed chronology of medical and pharmacy series used by each insured member over time.

Insurance benefits extend to all healthcare services. The HRI Database does not include data on clinical parameters (e.g., lab results), quality of life, or markers of disease severity because health insurers in Germany are prohibited by federal law from having such information. All patient-level data in the HRI Research Database are de-identified to comply with German data protection regulations. Use of the study database for health services research is therefore fully compliant with German federal law and, accordingly, IRB/ethical approval was not needed.

### Study population

The study population comprised persons who were enrolled in a health insurer represented in the HRI Database on January 1, 2009. Persons who were not continuously eligible for health insurance benefits for at least one year prior to January 1, 2009 were excluded from the study population; less than 0.5 % of the population from the HRI Database was excluded due to this criterion. Infants <12 months of age as of this date were not subject to this exclusionary criterion.

Study subjects were stratified based on their age (<5, 5–17, 18–49, 50–59, and ≥60 years) and risk profile (“at-risk”, “high-risk”, and “healthy”). Risk profiles were defined based on the presence of medical conditions that are indications for pneumococcal vaccination among children and adults including alcoholism, asthma, chronic heart disease, chronic liver disease, chronic lung disease, chronic renal failure, cochlear implant, Crohn’s disease, diabetes, Down’s syndrome, functional/anatomic asplenia, HIV, immunosuppressant therapy, short gestation/low birthweight, smoking, as well as those medical conditions hypothesized to be associated with increased risk including neuromuscular/seizure disorders, rheumatoid arthritis, and systemic lupus erythematosis [13–16]. The list of medical conditions was based on recommendations for vaccination set forth by STIKO and/or ACIP, as well as other conditions previously reported to confer an increased risk of pneumococcal disease.

Immunocompetent persons with ≥1 chronic medical condition were classified as at-risk; immunocompromised/immunosuppressed persons and those with a cochlear implant were classified as high-risk. At-risk and high-risk were mutually exclusive categories and thus, for example, persons considered immunosuppressed due to cancer treatment were included in the high-risk category only, even if they also had an at-risk condition. Persons without evidence of at-risk or high-risk conditions were classified as healthy. At-risk and high-risk medical conditions were ascertained using ICD-10-GM diagnosis codes recorded any time prior to the beginning of the 2009 calendar year. Operational algorithms employed to identify at-risk and high-risk conditions are available in Additional file 1: Table S1 and Additional file 2: Table S2, respectively, of the online supplement.

### Study measures

Episodes of all-cause pneumonia (i.e., all clinical cases caused by all known and unknown pathogens, including *S. pneumoniae*) were identified during the four-year period beginning on January 1, 2009 and ending on December 31, 2012 or the date of health insurer disenrollment, whichever occurred first. Episodes were identified using operational algorithms based on corresponding diagnosis codes (ICD-10-GM) in the principal or secondary position, procedure codes for inpatient care (Operationen- und Prozedurenschlüssel [OPS]), and ATC drug codes, as set forth in the online supplement (Additional file 3: Table S3); cases that were invasive (i.e., bacteremic) in nature were excluded from consideration. Multiple episodes of all-cause pneumonia for a given patient were included as independent events if they were separated by ≥90 days.

### Statistical analyses

Incidence rates of all-cause pneumonia episodes were estimated for children and adults within each age group by risk profile as well as individual medical condition, and were expressed per 100,000 person-years. Rate ratios for disease episodes among persons with at-risk and high-risk conditions, respectively—overall and by individual medical condition—versus their age-stratified healthy counterparts were estimated using Poisson regression (SAS v 9.3). Rates of disease and corresponding rate ratios (vs. healthy counterparts) also were calculated for at-risk persons by the number of at-risk conditions.

## Results

### Children

#### Characteristics

Children aged <5 years and 5–17 years contributed a total of 0.5 million and 1.7 million person-years of observation, respectively. In these two age groups, 71 % and 79 %, respectively, had none of the selected chronic or immunocompromising conditions, 26 % and 19 % had ≥1 at-risk condition (and no high-risk conditions), and 3 % and 2 % had a high-risk condition.

Among those with at-risk conditions, chronic lung disease (68 % and 54 %) and asthma (23 % and 43 %) were common; 17 % of children <5 years of age and 13 % of children 5–17 years of age with at-risk conditions had more than one condition.

#### Disease rates

**Fig. 1:**
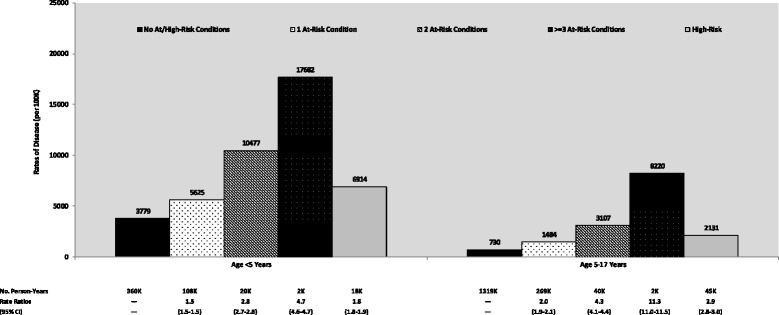
Rates of all-cause pneumonia among children by number of risk conditions

Rates of all-cause pneumonia among children <5 years and 5–17 years of age with at-risk conditions were 1.7 (95 % confidence interval [CI] 1.7-1.8) and 2.4 (2.3-2.5) times the rates in their healthy counterparts, and rates of all-cause pneumonia among high-risk children in these age groups were 1.8 (1.8-1.9) and 2.9 (2.8-3.0) times the rates in children without at-risk or high-risk conditions (Table 1). Rate ratios for all-cause pneumonia among children with at-risk conditions increased with the number of such conditions compared with healthy counterparts (Fig. 1). Among younger children, rate ratios increased from 1.5 (1.5-1.5) for those with one condition to 4.7 (4.6-4.7) for those with ≥3 conditions; among older children, rate ratios increased from 2.0 (1.9-2.1) to 11.3 (11.0-11.5).

**Table 1:** Rates of all-cause pneumonia among healthy, at-risk, and high-risk children

			All-Cause Pneumonia			
	No. of Person-Years		Age <5 Years		Age 5–17 Years	
	Age	Age	Rate	Rate Ratios^a^	Rate	Rate Ratios^a^
	<5 Years	5-17 Years	per 100 K	(95 % CI)	per 100 K	(95 % CI)
Risk Group						
Healthy	360,184	1,318,738	3,779	1.0	730	1.0
At-Risk	129,895	310,546	6,555	1.7 (1.7, 1.8)	1,734	2.4 (2.3, 2.5)
Chronic heart disease	15,181	19,899	7,813	2.1 (2.0, 2.1)	2,055	2.8 (2.7, 2.9)
Chronic lung disease	88,323	169,241	6,539	1.7 (1.7, 1.8)	1,836	2.5 (2.4, 2.6)
Diabetes	680	5,595	5,886	1.6 (1.5, 1.6)	1,734	2.4 (2.3, 2.5)
Asthma	29,737	134,188	8,986	2.4 (2.3, 2.4)	1,994	2.7 (2.6, 2.9)
Alcoholism	0	2,092	0	0.0 (----)	813	1.1 (1.0, 1.2)
Chronic liver disease	241	2,074	14,099	3.7 (3.7, 3.8)	2,941	4.0 (3.9, 4.2)
Smokers	0	530	0	0.0 (----)	1,321	1.8 (1.7, 1.9)
Down's syndrome	473	1,318	14,579	3.9 (3.8, 3.9)	4,022	5.5 (5.3, 5.7)
Neuromuscular/seizure disorder	9,075	18,219	7,570	2.0 (2.0, 2.0)	2,898	4.0 (3.8, 4.1)
Short gestation/low birthweight	10,311	32	8,719	2.3 (2.3, 2.4)	6,250	8.6 (8.4, 8.8)
Rheumatoid	67	1,250	10,448	2.8 (2.7, 2.8)	960	1.3 (1.2, 1.4)
Rheumatoid arthritis	8	358	0	0.0 (----)	280	0.4 (0.3, 0.4)
Lupus	0	59	0	0.0 (----)	0	0.0 (----)
Crohn's	59	841	11,865	3.1 (3.1, 3.2)	1,307	1.8 (1.7, 1.9)
High-Risk	17,704	45,383	6,914	1.8 (1.8, 1.9)	2,131	2.9 (2.8, 3.0)
Cochlear implant	1,134	2,684	5,907	1.6 (1.5, 1.6)	2,906	4.0 (3.8, 4.1)
Functional/anatomic asplenia	850	2,347	9,175	2.4 (2.4, 2.5)	2,983	4.1 (3.9, 4.2)
HIV	318	1,287	3,769	1.0 (1.0, 1.0)	1,710	2.3 (2.2, 2.5)
Chronic renal failure	1,127	4,579	12,778	3.4 (3.3, 3.4)	3,036	4.2 (4.0, 4.3)
Immunosuppressants	905	4,893	9,720	2.6 (2.5, 2.6)	1,696	2.3 (2.2, 2.4)
Malignant neoplasms	854	4,669	7,263	1.9 (1.9, 2.0)	1,328	1.8 (1.7, 1.9)
Solid organ transplantation	76	326	34,354	9.1 (9.0, 9.2)	6,441	8.8 (8.6, 9.0)
Congenital immunodeficiency	13,227	29,420	6,328	1.7 (1.6, 1.7)	2,094	2.9 (2.7, 3.0)
Diseases of white blood cells	683	1,468	11,267	3.0 (2.9, 3.0)	2,247	3.1 (3.0, 3.2)

^a^Relative to healthy counterparts

### Adults

#### Characteristics

Persons aged 18–49 years, 50–59 years, and ≥60 years contributed a total of 5.7 million, 2.0 million, and 3.5 million person-years of observation, respectively. Approximately 76 %, 60 %, and 36 %, respectively, had none of the selected chronic or immunocompromising conditions. The prevalence of at-risk and high-risk conditions increased with increasing age: 21 %, 33 %, and 45 % had at-risk conditions, and 3 %, 7 %, and 19 % had high-risk conditions.

Among adults aged 18–49 years with at-risk conditions, the most common conditions were chronic lung disease (48 %), asthma (27 %), and chronic liver disease (13 %). In adults 50–59 years of age, the most common conditions were chronic lung disease (37 %), diabetes (26 %), chronic liver disease (24 %), and chronic heart disease (23 %). In adults ≥60 years of age, the most common conditions were chronic heart disease (46 %), diabetes (41 %), chronic lung disease (30 %), and chronic liver disease (20 %).

#### Disease rates

Adults aged 18–49, 50–59, and ≥60 years with at-risk conditions had 2.2 (2.1-2.4), 2.3 (2.2-2.4), and 2.5 (2.4-2.5) times the rates of all-cause pneumonia as their healthy counterparts (Table 2). Corresponding rate ratios for those with high-risk conditions were 3.2 (3.0-3.4), 3.7 (3.6-3.9), and 4.1 (4.0-4.2).

**Table 2:** Rates of all-cause pneumonia among healthy, at-risk, and high-risk adults

				All-Cause Pneumonia					
	No. of Person-Years			Age 18–49 Years		Age 50–59 Years		Age ≥60 Years	
	Age	Age	Age	Rate	Rate Ratios^a^	Rate	Rate Ratios^a^	Rate	Rate Ratios^a^
	18-49 Years	50-59 Years	≥60 Years	per 100 K	(95 % CI)	per 100 K	(95 % CI)	per 100 K	(95 % CI)
Risk Group									
Healthy	4,334,344	1,194,431	1,308,873	441		684		1,439	
At-Risk	1,197,718	648,276	1,575,635	975	2.2 (2.1, 2.4)	1,554	2.3 (2.2, 2.4)	3,547	2.5 (2.4, 2.5)
Chronic heart disease	116,350	145,449	703,271	1,147	2.6 (2.5, 2.8)	1,780	2.6 (2.5, 2.7)	4,451	3.1 (3.0, 3.2)
Chronic lung disease	579,980	238,260	466,695	1,088	2.5 (2.3, 2.6)	2,195	3.2 (3.1, 3.3)	5,246	3.6 (3.5, 3.7)
Diabetes	97,028	165,443	643,437	1,115	2.5 (2.4, 2.7)	1,592	2.3 (2.2, 2.4)	3,736	2.6 (2.5, 2.7)
Asthma	323,267	102,513	165,056	1,112	2.5 (2.4, 2.7)	2,314	3.4 (3.2, 3.5)	4,621	3.2 (3.1, 3.3)
Alcoholism	33,593	25,488	27,142	1,578	3.6 (3.4, 3.8)	2,754	4.0 (3.9, 4.2)	5,626	3.9 (3.8, 4.0)
Chronic liver disease	154,804	154,616	321,444	1,015	2.3 (2.2, 2.5)	1,419	2.1 (2.0, 2.2)	2,861	2.0 (1.9, 2.1)
Smokers	38,316	22,141	19,819	1,133	2.6 (2.4, 2.7)	2,529	3.7 (3.6, 3.8)	5,535	3.8 (3.7, 3.9)
Down's syndrome	2,926	451	139	2,016	4.6 (4.4, 4.8)	7,987	11.7 (11.4, 11.9)	11,471	8.0 (7.8, 8.1)
Neuromuscular/seizure disorder	49,368	18,445	36,733	1,669	3.8 (3.6, 4.0)	2,483	3.6 (3.5, 3.8)	6,711	4.7 (4.6, 4.8)
Short gestation/low birthweight	1,607	0	4	622	1.4 (1.3, 1.5)	0	0.0 (----)	0	00.0 (----)
Rheumatoid	48,367	35,563	83,318	1,164	2.6 (2.5, 2.8)	1,696	2.5 (2.4, 2.6)	3,842	2.7 (2.6, 2.8)
Rheumatoid arthritis	26,323	28,126	76,319	1,193	2.7 (2.6, 2.9)	1,582	2.3 (2.2, 2.4)	3,830	2.7 (2.6, 2.7)
Lupus	1,624	794	1,028	2,402	5.5 (5.2, 5.7)	3,777	5.5 (5.3, 5.7)	5,640	3.9 (3.8, 4.0)
Crohn's	21,202	7,128	6,633	990	2.2 (2.1, 2.4)	1,796	2.6 (2.5, 2.7)	3,317	2.3 (2.2, 2.4)
High-Risk	181,558	132,436	630,789	1,411	3.2 (3.0, 3.4)	2,559	3.7 (3.6, 3.9)	5,852	4.1 (4.0, 4.2)
Cochlear implant	719	542	2,787	1,669	3.8 (3.6, 4.0)	2,768	4.0 (3.9, 4.2)	4,629	3.2 (3.1, 3.3)
Functional/anatomic asplenia	11,350	3,580	8,583	1,665	3.8 (3.6, 4.0)	4,134	6.0 (5.9, 6.2)	9,705	6.7 (6.6, 6.9)
HIV	7,940	2,692	7,386	1,952	4.4 (4.2, 4.6)	2,192	3.2 (3.1, 3.3)	3,994	2.8 (2.7, 2.9)
Chronic renal failure	38,170	36,522	266,953	1,776	4.0 (3.8, 4.2)	3,313	4.8 (4.7, 5.0)	8,114	5.6 (5.5, 5.8)
Immunosuppressants	84,727	81,587	381,069	1,779	4.0 (3.9, 4.2)	2,787	4.1 (3.9, 4.2)	5,053	3.5 (3.4, 3.6)
Malignant neoplasms	82,755	80,199	379,086	1,556	3.5 (3.4, 3.7)	2,557	3.7 (3.6, 3.9)	4,957	3.4 (3.4, 3.5)
Solid organ transplantation	2,574	1,850	2,924	8,509	19.3 (18.9, 19.7)	12,056	17.6 (17.3, 17.9)	15,768	11.0 (10.8, 11.1)
Congenital immunodeficiency	38,927	10,800	16,179	1,123	2.5 (2.4, 2.7)	2,935	4.3 (4.1, 4.4)	6,385	4.4 (4.3, 4.5)
Diseases of white blood cells	9,468	5,779	13,734	1,986	4.5 (4.3, 4.7)	3,063	4.5 (4.3, 4.6)	6,407	4.5 (4.3, 4.6)

^a^Relative to healthy counterparts

**Fig. 2:**
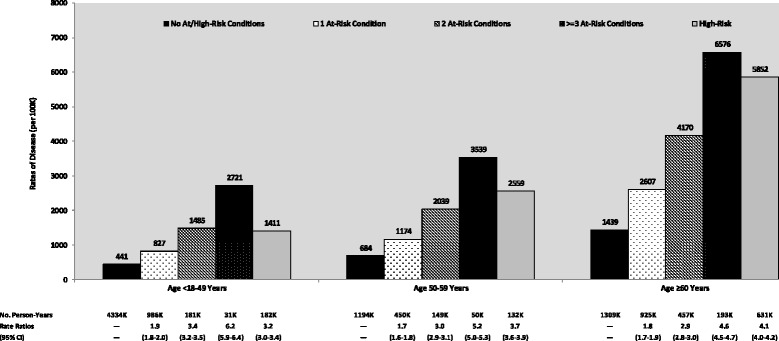
Rates of all-cause pneumonia among adults by number of risk conditions

Rate ratios for all-cause pneumonia among adults with at-risk conditions increased with the number of such conditions compared with healthy counterparts (Fig. 2). Rates among adults with two at-risk conditions were generally similar to rates among adults with high-risk conditions, and rates in adults with three or more at-risk conditions were higher than those among adults with high-risk conditions. Rates and rate ratios of all-cause pneumonia among healthy, at-risk, and high-risk adults stratified by additional age groups are available in Additional file 4: Table S4 of the online supplement.

## Discussion

We undertook a large retrospective evaluation of rates of all-cause pneumonia among “at-risk” and “high-risk” children and adults in Germany—in comparison with age-stratified healthy counterparts—in the period following the 2006 recommendation for universal immunization of infants with pneumococcal conjugate vaccine. The results of this evaluation suggest that immunocompetent persons with comorbid conditions and immunocompromised persons of all ages continue to suffer a disproportionate burden of pneumonia in the era of widespread pneumococcal vaccination [3, 17–21]. Both the proportion of the German population with an at-risk or high-risk condition and the incidence of CAP are reported to climb with increasing age. Moreover, the results of this study suggest that the risk of all-cause pneumonia among children and adults with ≥2 at-risk conditions is comparable to, or exceeds, corresponding values among age-stratified persons with high-risk conditions. Our data also provide additional evidence in support of an increased risk of all-cause pneumonia in adults with rheumatoid arthritis, systemic lupus erythematosus, Crohn’s disease, and neuromuscular/seizure disorders [13, 22]. Such conditions are not currently included within the STIKO or ACIP recommendations for prevention.

Notwithstanding differences in study design and methods between our evaluation and work by Ewig and colleagues—including their use of records from all hospitals in Germany (vs. claims information from an age/gender-representative sample in our study), their focus on an adult population (vs. children and adults), and their focus on inpatient CAP (vs. inpatient and outpatient all-cause pneumonia)—the results of our study are largely consistent with their findings. Ewig et al. identified cardiac, central nervous system, and pulmonary disease (other than COPD), as well as diabetes and COPD, as the five most prevalent comorbid conditions in adults with hospitalized CAP. Similarly, we found chronic lung disease, chronic heart disease, and diabetes to be the most prevalent chronic diseases among Germans with all-cause pneumonia; asthma and chronic liver disease also were found to be common among pneumonia cases.

Recent studies from the UK and US found that the elevated risk of pneumonia in adults with selected medical conditions persisted during the era following the introduction of pneumococcal conjugate vaccine, although the magnitude of relative risk was not always consistent [13, 22–24]. In our study, German children under the age of 5 years with at-risk conditions accounted for 26 % of this age cohort yet 36 % of all-cause pneumonia cases; children aged 5–17 years with at-risk conditions accounted for 19 % of this cohort but 34 % of all-cause pneumonia cases. Comparable results for adults aged <60 years were: 18–49 year-olds, 21 % of the cohort and 35 % of disease burden; 50–59 year-olds, 33 % of the cohort and 47 % of disease burden. Consistent with published data from Shea and colleagues, we found increasing incidence of pneumonia with increasing age in at-risk, high-risk, and healthy persons, and relatively stable rate ratios, suggesting that the relative increase in disease risk with age is similar in all three groups [13].

We chose all-cause pneumonia as a proxy for pneumococcal pneumonia based on several considerations. First, studies in children demonstrate that identification of pneumococcus as a cause of hospitalized pneumonia substantially underestimates the role of pneumococcus compared to the reduction in all-cause hospitalized pneumonia achieved following introduction of pneumococcal conjugate vaccine [25, 26]. We believe the same challenge would be relevant for the diagnosis of pneumococcal pneumonia in adults in clinical settings. Moreover, operational algorithms for pathogen-specific cases of pneumonia based on diagnosis codes typically lack adequate sensitivity since diagnostic tests (i.e., culture, serological, and polymerase chain reaction [PCR] tests, as well as invasive sampling methods) are infrequently performed in clinical practice. Thus, in the absence of concurrent bacteremia, the diagnosis (and therefore coding) of pneumococcal pneumonia underestimates disease burden. Second, in the control group in the CAPiTA study, 22 % (174 of 787) of episodes of CAP in adults ≥65 years of age were due to vaccine serotypes representing a minimum estimate of pneumococcal burden in this age group as nonvaccine serotypes were not sought. Lastly, in the retrospective study by Shea and colleagues based on US healthcare claims data, the increased disease risk in “at-risk” and “high-risk” subjects was comparable for both all-cause pneumonia and pneumococcal pneumonia [13].

We note several other limitations that are inherent in the use of healthcare claims data for retrospective studies such as this one. First, rates of all-cause pneumonia may be misestimated somewhat due to the less than perfect sensitivity and specificity of our case-ascertainment algorithm. However, to the extent this limitation impacts rates in a proportional manner across age and risk groups, rate ratios should be largely unaffected. Second, use of operational algorithms and the left-truncation of the study database undoubtedly resulted in the misclassification of risk profiles for some persons, including both errors of omission and commission. For example, diagnosis codes capturing smoking and alcoholism are under-recorded in the study database, and procedures (e.g., cochlear implant) performed prior to the beginning of the study database were not observable. Unfortunately, it was not possible to undertake a formal evaluation—for example, via chart review or use of additional data sources (e.g., electronic medical records)—of the accuracy of these algorithms within the context of this study. Third, data limitations precluded us from identifying the specific pathogen/serotype causing disease; it would be of interest to know the proportion of cases due to individual pathogens/serotypes, and whether or not they are included in PCV7, PCV10, PCV13, and PPSV23 (or currently unavailable vaccines). Fourth, to the extent pneumococcal vaccination status varies by risk profile (i.e., likely to be higher uptake among high-risk and at-risk persons vs. healthy persons), rate ratios may be downwardly biased. Fifth, while the HRI Database comprises claims data from individuals covered by the German statutory health care system and is representative of the German population in terms of age and gender, it is unknown whether the HRI Database is representative of the German population in terms of race, socio-economic factors, and other such characteristics. Finally, while the HRI Research Database should be sufficiently large to compare (robustly) rates of disease between age-specific at-risk and high-risk persons versus their healthy counterparts, comparisons of rates within subgroups defined on the basis of individual conditions are undoubtedly underpowered in many instances and should be interpreted with caution.

## Conclusion

Our data confirm previous reports of enhanced risk in individuals with multiple comorbid conditions [13, 22, 27]. Expanded use of pneumococcal conjugate vaccines have been proposed to prevent pneumococcal pneumonia in high-risk individuals and those 65 years of age and older. In conjunction with recent publications [11, 13, 22, 23, 28], our findings suggest that the highest rates of all-cause pneumonia, and presumably pneumococcal pneumonia, occur in individuals with multiple comorbid conditions in the absence of immune deficiency. In adults with traditional high-risk conditions, high mortality rates have been reported [3]. If high mortality rates from all-cause pneumonia and pneumococcal pneumonia also are observed in individuals with multiple at-risk conditions in the absence of immune compromise, further effort to understand the underlying susceptibility could lead to additional strategies for prevention.

## Additional files

Additional file 1: Table S1. Diagnosis, procedure, and drug codes for algorithms identifying at-risk conditions. (DOC 91 kb) Additional file 2: Table S2. High risk-conditions: Diagnosis, procedure, and drug codes for algorithms identifying baseline risk factors and study measures. (DOC 70 kb) Additional file 3: Table S3. Outcome: Diagnosis, procedure, and drug codes for algorithms identifying all-cause pneumonia *. (DOC 97 kb) Additional file 4: Table S4. Rates of all-cause pneumonia among healthy, at-risk, and high-risk adults, by age. (DOC 941 kb)

## Abbreviations

ATC: Anatomic Therapeutic Chemical
COPD: Chronic obstructive pulmonary disease
CAP: Community-acquired pneumonia
HRI: Health Risk Institute
ICD-10-GM: International Statistical Classification of Diseases and Related Health Problem, 10^th^ revision, German Modification
OPS: Operationen- und Prozedurenschlüssel
OPS: Polymerase Chain Reaction
PCR: Pneumococcal conjugate vaccine
PCV7: Seven-valent pneumococcal conjugate vaccine
STIKO: Ständige Impfkommission
pneumococcus: *Streptococcus pneumoniae*
PCV10: Ten-valent pneumococcal conjugate vaccine
PCV13: Thirteen-valent pneumococcal conjugate vaccine
PPSV23: Twenty-three-valent pneumococcal polysaccharide vaccine
ACIP: US Advisory Committee for Immunization Practices
