# Supplementary material for: Rates of pneumonia among children and adults with chronic medical conditions in Germany
Source: BMC Infect Dis. 2015 Oct 30;15:470. doi: 10.1186/s12879-015-1162-y (PMC4627378; doi:10.1186/s12879-015-1162-y)
Supplement: Supplementary file 1 [file 12879_2015_1162_MOESM1_ESM.doc]

Table S1. Diagnosis, procedure, and drug codes for algorithms identifying at-risk conditions

	ICD-10-GM 2008			
**Condition**	**Code**	**Description (English)**	**Description (German)**	**Prescriptions**
Chronic Heart Disease	I05.*-I09.*	Chronic rheumatic heart disease	Chronische rheumatische Herzkrankheiten	
	I11.*	Hypertensive heart disease	I11 Hypertensive Herzkrankheit (I11.0 Hypertensive Herzkrankheit mit (kongestiver) Herzinsuffizienz / I11.9 Hypertensive Herzkrankheit ohne (kongestive) Herzinsuffizienz)	
	I13.*	Hypertensive heart and chronic kidney disease	Hypertensive Herz- und Nierenkrankheit	
	I21.*, I22.*	I21 ST elevation (STEMI) and non-ST elevation (NSTEMI) myocardial infarction; I22 Subsequent ST elevation (STEMI) and non-ST elevation (NSTEMI) myocardial infarction	Akuter/ rezidivierender Myokardinfarkt	
	I24.*	Other acute ischemic heart disease	Sonstige akute ischämische Herzkrankheit	Prior
	I25.2	Old myocardial infarction	Alter Myokardinfarkt	≥1 verified
	I20.*	I20.x Includes	Angina pectoris	
	I25.1	Atherosclerotic heart disease of native coronary artery	Atherosklerotische Herzkrankheit	≥1 inp
	I27.*	Other pulmonary heart disease	Sonstige pulmonale Herzkrankheit	
	I34.*-I39.*	I34 nonrheumatic mitral valve disorders; I35 nonrheumatic aortic valve disorders; I36 nonrheumatic tricuspid valve disorders; I37 nonrheumatic pulmonary valve disorders; I38 endocarditis, valve unspecified; I39 endocarditis and heart valve disorders in diseases classified elsewhere	I34 Nichtrheumatische Mitralklappenkrankheiten<br>I35 Nichtrheumatische Aortenklappenkrankheiten<br>I36 Nichtrheumatische Trikuspidalklappenkrankheiten<br>I37 Pulmonalklappenkrankheiten<br>I38 Endokarditis, Herzklappe nicht näher bezeichnet<br>I39 Endokarditis und Herzklappenkrankheiten bei anderenorts klassifizierten Krankheiten	=> Outpa<br>Germany,<br>quarter ca<br>the Ge<br>reimburse<br>no exac<br>available<br>can be c
	I42.*	I42 cardiomyopathy	Kardiomyopathie	
	I50.*	398.91 and I09.81 Rheumatic heart failure	Herzinsuffizienz	
	I23.*	Certain current complications following ST elevation (STEMI) and non-ST elevation (NSTEMI) myocardial infarction (within the 28 day period)	Bestimmte akute Komplikationen nach akutem Myokardinfarkt	=> Only d<br>"verifi
	Q25.1	Coarctation of aorta	Koarktation der Aorta	
	Q20.*-Q24:*	Q20 Congenital malformation of cardiac chambers and connections; Q21 Congenital malformations of cardiac septa; Q22 Congenital malformations of pulmonary and tricuspid valves; Q23 Congenital malformations of aortic and mitral valves; Q24 Other congenital malformations of heart	Q20 Angeborene Fehlbildungen der Herzhöhlen und verbindender Strukturen<br>Q21 Angeborene Fehlbildungen der Herzsepten<br>Q22 Angeborene Fehlbildungen der Pulmonal- und Trikuspidalklappe<br>Q23 Angeborene Fehlbildungen der Aorten- und der Mitralklappe<br>Q24 Sonstige angeborene Fehlbildungen des Herzens	
Chronic lung disease	E84.*	Cystic fibrosis	Zystische Fibrose	
	I27.8	Other specified pulmonary heart disease	Sonstige näher bezeichnete pulmonale Herzkrankheiten	
	I27.9	Pulmonary heart disease unspecified	Pulmonale Herzkrankheiten, nicht näher bezeichnet	
	J40	Bronchitis, not specified as acute or chronic	Bronchitis, nicht als akut oder chronisch bezeichnet	
	J41,.* J42	J41 Simple and mucopurulent chronic bronchitis; J42 Unspecified chronic bronchitis	J41 Einfache und schleimig-eitrige chronische Bronchitis<br>J42 Nicht näher bezeichnete chronische Bronchitis	
	J43.*	Emphysema	Emphysem	
	J47	Bronchiectasis	Bronchiektasien	
	J44.*	Other chronic obstructive pulmonary disease	Sonstige chronische obstruktive Lungenkrankheit	Prior
	J60.*-J65.*	J60 Coalworker's pneumoconiosis; J61 Pneumoconiosis due to dust containing silica; J63 Pneumoconiosis due to other inorganic dusts; J64 Unspecified pneumoconiosis; J65 Pneumoconiosis associated with tuberculosis	J60 Kohlenberarbeiter-Pneumokoniose; J61 Pneumokoniose durch Asbest und sonstige anorganische Fasern; J62 Pneumokoniose durch Quarzstaub; J63 Pneumokoniose durch sonstige anorganische Stäube; J64 Nicht näher bezeichnete Pneumokoniose; J65 Pneumokoniose in Verbindung mit Tuberkulose	≥1 verified<br>≥1 inp
	J68..4	Chronic respiratory conditions due to inhalation of chemicals, gases, fumes and vapors	Chronische Krankheiten der Athmungsorgane durch chemischen Substanzen, Gase, Rauch und Dämpfe	
	J84.1	Pulmonary fibrosis, unspecified (includes postinflammatory pulmonary fibrosis)	Sonstige interstitielle Lungenkrankheiten mit Fibrose	
	J84.*	Other interstitial pulmonary diseases	Sonstige interstitielle Lungenkrankheiten	
	J96.1	Chronic respiratory failure	Chronische respiratorische Insuffizienz, andererorts nicht klassifiziert	
	J96.*	Acute and chronic respiratory failure	Respiratorische Insuffizienz, andernorts nicht klassifiziert	
	P27.*	Chronic respiratory disease originating in the perinatal period	Chronische Atemwegskrankheit mit Ursprung in der Perinatalperiode	
Asthma	J45.*, J46		J45 Asthma bronchiale; J46 Status asthmaticus	[illegible]<br>≥1 verified<br>≥1 inp
Diabetes	E10.*-E14.*	Diabetes mellitus	Diabetes mellitus	
	E10.4, E11.4. E12.4, E13.4. E14.4		Diabetes mit neurologischen Komplikationen: Polyneuropathie	
	E10.3, E11.3, E12.3, E13.3, E14.3		Diabetes mit Augenkomplikationen: Retinopathie	
	E10.3, E11.3, E12.3, E13.3, E14.3		Diabetes mit Augenkomplikationen: Katarakt	
	P70.1	Syndrome of infant of a diabetic mother	P70.1 Syndrom des Kindes einer diabetischen Mutter	
	T38.3		Insulin und orale blutzuckersenkende Arzneimittel	
	Manifestationen G59.0*, G63.2*	Diabetic mononeuropathy	G59.0* Diabetische Mononeuropathien (*Organmanifestation)	
		Diabetic polyneuropathy	G63.2* Diabetische Polyneuropathien (*Organmanifestation)	
Alcoholism	F10.0, F10.5, F10.6, F10.7, F10.8, F10.9		F10.0 Akute Intoxikation<br>F10.5 Psychotische Störung<br>F10.6 Amnestisches Syndrom<br>F10.7 Restzustand und verzögert auftretende psychotische Störung<br>F10.8 Sonstige psychische und Verhaltensstörungen<br>F10.9 Nicht näher bezeichnete psychische und Verhaltensstörungen	Prior to s<br>><br>≥1 ve<br>c<br>≥1 inp
	F10.2	Alcohol dependence	F10.2 Psychische und Verhaltensstörungen durch Alkohol - Abhängigkeitssyndrom	
Chronic liver disease	K70.*, K70.0, K70.1, K70.3, K70.9, K73.*, K74.*, K74.5, K76.*	K70 Alcoholic liver disease, K70.0 Alcoholic fatty liver, K70.1 Alcoholic hepatitis, K70.3 Alcoholic cirrhosis of liver, K70.9 Alcoholic liver disease, unspecified K73 Chronic hepatitis not elsewhere classified, K74 Fibrosis and cirrhosis of liver, K74.5 Biliary cirrhosis, unspecified, K76 Other diseases of liver	K70 Alkoholische Leberkrankheit<br>K70.0 Alkoholische Fettleber; K70.1 Alkoholische Hepatitis; K70.3 Alkoholische Leberzirrhose; K70.9 Alkoholische Leberkrankheit, nicht näher bezeichnet; K73 Chronische Hepatitis, andernorts nicht klassifiziert K74 Fibrose und Zirrhose der Leber K74.5 Biliäre Zirrhose, nicht näher bezeichnet. K76 Sonstige Krankheiten der Leber	Prior<br>≥1 verified
	K72.7*, K75.0, K75.1, K76,6, K76.7	K72.X1 Hepatic failure with coma; K75.0 Abscess of liver; K75.1 Phlebitis of portal vein; K76.6 Portal hypertension; K76.6 Hepatorenal syndrome	K72.7* Hepatische Enzephalopathie und Coma hepaticum<br>K75.0 Leberabszess<br>K75.1 Phlebitis der Pfortader<br>K76.6 Portale Hypertonie<br>K76.7 Hepatorenales Syndrom	≥1 inp
	K76.1		Chronische Stauungsleber	
	I85.0		Ösophagusvarizen mit Blutung	
	I85.9		Ösophagusvarizen ohne Blutung	

Table S1. Diagnosis, procedure, and drug codes for algorithms identifying at-risk conditions (Cont.)

	ICD-10-GM 2008				
**Condition**	**Code**	**Description (English)**	**Description (German)**	**Prescriptions**	[illegible]
Smokers	F17.2	Nicotine dependence	Psychische und Verhaltensstörungen durch Tabak (Abhängigkeitssyndrom)		Prior to s[illegible]<br>>[illegible]<br>≥1 verified [illegible]<br>≥1 inp[illegible]
Down's Syndrome	Q90.*	Down syndrome	Down-Syndrom		Prior [illegible]<br>≥1 ver[illegible]<br>d[illegible]<br>≥1 inp[illegible]
Neuromuscular Disorders	G24.0, G24.2, G80.3	G80.3 Athetoid cerebral palsy; G24.0 Drug-induced dystonia; G24.2 Idiopathic nonfamilial dystonia	G24.0 Arzneimittelinduzierte Dystonie<br>G24.2 Idiopathiosche nichtfamiliäre Dystonie<br>G80.3 Athetotische Zerebralparese		
	G80.*	Cerebral palsy	Infantile Zerebralparese		
	G40.*, G41.*	Epilepsy and recurrent seizures	G40 Epilepsie<br>G41 Status epilepticus		Prior [illegible]<br>≥1 ver[illegible]
	P90	Convulsions of newborn	Krämpfe bei Neugeborenen		d[illegible]
	R56	Convulsions not eslewhere classified	Krämpfe, anderenorts nicht klassifiziert		≥1 inp[illegible]
	G71.*, G72.*	Primary disorders of muscles	G70 Mysthenia gravis und sonstige neuromuskuläre Krankheiten<br>G71 Primäre Myopathien<br>G72 Sonstige Myopathien<br>G73.* Krankheiten im Bereich der neuromuskulären Synapse und des Muskels bei anderenorts klassifizierten Krankheiten		
Disorders relating to short gestation and low birthweight		P07.2 Extreme immaturity of newborn; P07.0 Extreme low birth weight newborn	P07.0 Neugeborenes mit extrem niedrigem Geburtsgewicht<br>P07.2 Neugeborenes mit extremer Unreife		Prior [illegible]<br>≥1 ver[illegible]
		P07.3 Preterm newborn: P07.1 Other low birth weight newborn	P07.1 Neugeborenes mit sonstigem niedrigem Geburtsgewicht<br>P07.3 Sonstige vor dem Termin Geborene		d[illegible]<br>≥1 inp[illegible]
Rheumatoid arthritis	M05.*, M06.*		M05 Seropositive chronische Polyarthritis<br>M06 Sonstige chronische Polyarthritis		
Lupus	M32.0, M32.1, M32.8, M32.9		M32 Systemischer Lupus erythematodes<br>M32.0 Arzneimittelinduzierter systemischer Lupus erythematodes<br>M32.1 Systemischer Lupus erythematodes mit Beteiligung von Organen oder Organsystemen<br>M32.8 Sonstige Formen des systemischen Lupus erythematodes<br>M32.9 Systemischer Lupus erthematodes, n.n.b.		Prior [illegible]<br>≥1 ver[illegible]<br>d[illegible]<br>≥1 inp[illegible]
Crohn's	K50.0, K50.1, K50.8, K50.9		K50 Crohn-Krankheit<br>K50.0 Crohn-Krankheit des Dünndarms<br>K50.1 Crohn-Krankheit des Dickdarms<br>K50.8 Sonstige Crohn-Krankheit<br>K50.9 Crohn-Krankheit, nicht näher bezeichnet		
