# Supplementary material for: Rates of pneumonia among children and adults with chronic medical conditions in Germany
Source: BMC Infect Dis. 2015 Oct 30;15:470. doi: 10.1186/s12879-015-1162-y (PMC4627378; doi:10.1186/s12879-015-1162-y)
Supplement: Supplementary file 2 [file 12879_2015_1162_MOESM2_ESM.doc]

Table S2. High-risk conditions: Diagnosis, procedure, and drug codes for algorithms identifying baseline risk factors and study measures

	ICD-10-GM (2008)				
Condition	Code	Description (English)	Description (German)	Prescriptions	Algorith
Functional or Anatomic Asplenia					For all conditions<br>≥1 verified outpatier<br>≥1 inpatient d<br><br>=> Outpatient diagnos<br>only one episode per<br>counted due to the Ge<br>reimbursement system.<br>date of diagnosis ava<br>diagnosis can be code<br>quarte<br>=> Only diagnoses mar<br>were app<br>=> Prescription has to<br>quarter of the verifi<br>diagnos
Asplenia	Q89.0	Q89.01 Asplenia (congenital); Z90.81 Acquired absence of spleen	Angeborene Fehlbildung der Milz (Asplenie (angeboren))<br>Verlust sonstiger Organe		
Sickle cell disease and other hemoglobinopathies	D56.*	Thalassemia	Thalassämie		
	D57.3	Sickle-cell trait	Sichelzellen-Erbanlage		
	D57.* (except D57.3, see Sickle-cell trait)	Sickle-cell disorders	Sichelzellenkrankheiten		
	D58.2	Other hemoglobinopathies	Sonstige Hämoglobinopathien		
	D60.*, D61.*	D60 Acquired pure red cell aplasia (erythroblastopenia); D61 Other aplastic anemias and other bone marrow failure syndromes	D60 Erworbene isoliere aplastische Anämie (Erythroblastopenie), D61 Sonstige aplastische Anämien		
	D73.1	Hypersplenism	Hyperspleni smus		
	D73.8	Other disease of spleen	Sonstige Krankheiten der Milz		
	Q89.9	Congental malformation, unspecified	Angeborene Fehlbildung, n.n.b.		
	Q89.3	Sits inversus	Situs inversus		
HIV infection	B20-B23.*, B24<br>Z21	B20 HIV disease; O98.7 HIV disease complicating pregnancy, childbirth, and puerperium; Z21 Asymptomatic HIV infection status	B20-B24 HIV-Krankheit<br>Z21 Asymptomatische HIV-Infektion		
Chronic renal failure			Chronische Nierenerkrankungen		
	I12.*	Hypertensive chronic kidney disease	Hypertensive Nierenkrankheit		
	I13	Hypertensive heart and chronic kidney disease	Hypertensive Herz- und Nierenkrankheit		
	N03	chronic nephritic syndrome	Chronisches nephritisches Syndrom - Glomerulonephritis		
	N04, N05	N04 nephritic syndrome, N05 Unspecified nephritic syndrome	N04 Nephrotisches Syndrom<br>N05 Nicht näher bezeichnetes nephritisches Syndrom		
	N00, N01, N03	Nephritic syndrome	N00 Akutes nephrisitsches Syndrom N01 Rapid-progressives nephritisches Syndrom N03 Chronisch nephritisches Syndrom		
	N18.*	Chronic kidney disease	Chronische Niereninsuffizienz		
	N19	Unspecified kidney failure	Nicht näher bezeichnete Niereninsuffizienz		
	Q60.*	Renal agenesis and other reduction defects of kidney	Nierenagenesie und sonstige Reduktionsdefekte der Niere		
	Z94.0	Kidney transplant status	Zustand nach Nierentransplantation		
	Z99.2	Z91.15 Patient's noncompliance with renal dialysis; Z99.2 Dependence on renal dialysis	Langzeitige Abhängigkeit von Dialyse bei Niereninsuffizienz		
	Z49	Encounter for care involving renal dialysis	Dialysebehandlung		
	Z49.2		Sonstige Dialyse - Peritonealdialyse		
	N04		Nephrotisches Syndrom		
Diseases associated w/ immuno Rx or radiation Tx					
Malignant neoplasms	C00-C97 (except C44)		Neoplastische Krankheiten		
	Z51.0	Encounter for antineoplastic radiation therapy	Strahlentherapie-Sitzung		
	Z51.1	Encounter for antineoplastic chemotherapy	Chemotherapie-Sitzung wegen bösartiger Neubildung		
			Strahlentherapie		
			Chemotherapie		
	Z51.5	Encounter for palliative care	Palliativbehandlung		
	Z08.*	Follow-up examination after completed treatment for malignant neoplasm	Nachuntersuchung nach Behandlung wegen bösartiger Neubildung		
Solid organ transplantation			(Zustand nach) Organ- oder Gewebetransplantation		
	Z94.6		Zustand nach Knochentransplantation		
	Z94.80, Z94.81		Z94.80 Z.n. hämatopoetischer Stammzelltransplantation ohne gegenwärtiger Immunsuppression<br>Z94.81 Z.n. hämatopoetischer Stammzelltransplantation mit gegenwärtiger Immunsuppression		
	Z94.0		Z. n. Nierentransplantation		
	Z94.1		Z. n. Herztransplantation		
	Z94.4		Z. n. Lebertransplantation		
	Z94.2		Z. n. Lungentransplantation		
	Z94.88		Z. n. sonstiger Organ- oder Gewebetransplantation		
	Z94.9		Z. n. Organ- oder Gewebetransplantation, nicht näher bezeichnet		
	Z94.9		Z. n. Organ- oder Gewebetransplantation, nicht näher bezeichnet		
	Z94.3	Heart and lungs transplant status	Z. n. Herz-Lungen-Transplantation		

Table S2. High-risk conditions: Diagnosis, procedure, and drug codes for algorithms identifying baseline risk factors and study measures (Cont.)

	ICD-10-GM (2008)				
**Condition**	**Code**	**Description (English)**	**Description (German)**	**Prescriptions**	**Algorith**
Congenital immunodeficiency	D80.*, D81.*, D82.*, D83.*, D84.*, D89.*	D80 Immunodeficiency with predominantly antibody defects; D81 Combined immunodeficiencies; D82 Immunodeficiency associated with other major defects; D83 Common variable immunodeficiency; D84 Other immunodeficiencies; D899 Other disorders involving the immune mechanism not elsewhere classified	D80 Immundefekt mit vorherrschendem Antikörpermangel<br>D81 Kombinierte Immundefekte<br>D82 Immundefekt in Verbindung mit anderen schweren Defekten<br>D83 Variabler Immundefekt<br>D84 Sonstige Immundefekte<br>D89 Sonstige Störungen mit Beteiligung des Immunsystems, anderenorts nicht klassifiziert		
Diseases of white blood cells	D70.*		Neutropenie		
	D71		Funktionelle Störung der neutrophilen Granulozyten		
Cochlear implant	Z96.2	Cochlear implant status	Vorhandensein von Implantaten im Gehörgang - Kochlearimplantat		
	5-209.2	Insertion of cochlear prothesis	Einführung eines Kochleaimplantates		
	5-209.7, 5-209.8		Wechsel, Entfernung eines Kochleaimplantates		
