# Supplementary material for: Rates of pneumonia among children and adults with chronic medical conditions in Germany
Source: BMC Infect Dis. 2015 Oct 30;15:470. doi: 10.1186/s12879-015-1162-y (PMC4627378; doi:10.1186/s12879-015-1162-y)
Supplement: Supplementary file 3 [file 12879_2015_1162_MOESM3_ESM.doc]

Table S3. Outcomes: Diagnosis, procedure, and drug codes for algorithms identifying all-cause pneumonia*

	ICD-10-GM (2009)				
**Condition**	**Code**	**Description (English)**	**Description (German)**	**Prescriptions**	**Episode**
Pneumonia				Antibiotics ATC-Codes (see S3-Guideline for Pneumonia 2009):<br>J01AA Tetracycline<br>J01CA Penicilline mit erweitertem Wirkungsspektrum<br>J01DC Cephalosporine der 2. Generation<br>J01DD Cephalosporine der 3. Generation<br>J01DE Cephalosporine der 4. Generation<br>J01DH Carbapeneme<br>J01FA Makrolide<br>J01MA Fluorchinolone<br>J05AB Nukleoside und Nekleotide	Criteria: Inpatient diagnosis or verified outpatient diagnosis + antibiotic prescription (in the same quarter) (Date of prescription was defined as index date of diagnosis) (Episodes must be 90 days apart)<br><br>Duration: Inpatient, length of hospital stay; outpatient episodes were truncated at 90 days after date of antibiotic prescription
All-Cause					
	J10.0		J10.0 Grippe mit Pneumonie, sonst. Influenzaviren nachgewiesen		
	J11.0		J11.0 Grippe mit Pneumonie, Viren nicht nachgewiesen		
	J12		J12 Viruspneumonie		
	J13		J13 Pneumonie durch Streptococcus pneumoniae		
	J14		J14 Pneumonie durch Haemophilus influenzae		
	J15		J15 Pneumonie durch Bakterien		
	J16		J16 Pneumonie durch sonstige Infektionserreger		
	J17*		J17* Pneumonie bei anderenorts klassifizierten Krankheiten (*Organmanifestation)		
	J18		J18 Pneumonie, Erreger n.n.b.		
	J86	J86 Pyothorax	Pyothorax		
Pneumococcal	J13		Pneumonie durch Streptococcus pneumoniae		
	J15.9 + B95.3		Bakterielle Pneumonie, n.n.b. + Streptococcus pneumoniae		
	J18.0 + B95.3		Bronchopneumonie, nicht näher bezeichnet + Streptococcus pneumoniae		
	J18 + B95.3		Pneumonie, Erreger n.n.b. + Streptococcus pneumoniae		
	J86 + B95.3		Pyothorax (Empyem) + Streptococcus pneumoniae		

*Cases of invasive disease, irrespective of pathogen, were excluded from analysis
