# Supplementary material for: Rates of pneumonia among children and adults with chronic medical conditions in Germany
Source: BMC Infect Dis. 2015 Oct 30;15:470. doi: 10.1186/s12879-015-1162-y (PMC4627378; doi:10.1186/s12879-015-1162-y)
Supplement: Supplementary file 4 [file 12879_2015_1162_MOESM4_ESM.doc]

Table S4: Rates of all-cause pneumonia among healthy, at-risk, and high-risk adults, by age

	No. of Person-Years						Age 18-49 Years		Age 50-5
	Age 18-49 Years	Age 50-59 Years	Age 60-64 Years	Age 65-74 Years	Age 75-84 Years	Age ?85 Years	Rate per 100K	Rate Ratios (95% CI)	Rate per 100K
**Risk Group**									
**Healthy**	4,334,344	1,194,431	362,456	664,997	233,248	48,172	441	---	684
**At-Risk**	1,197,718	648,276	304,618	761,822	408,353	100,843	975	2.2 (2.1, 2.4)	1,554
Chronic heart disease	116,350	145,449	94,143	314,648	226,558	67,922	1,147	2.6 (2.5, 2.8)	1,780
Chronic lung disease	579,980	238,260	97,815	223,093	119,044	26,742	1,088	2.5 (2.3, 2.6)	2,195
Diabetes	97,028	165,443	111,004	314,523	176,728	41,183	1,115	2.5 (2.4, 2.7)	1,592
Asthma	323,267	102,513	40,965	85,284	33,477	5,330	1,112	2.5 (2.4, 2.7)	2,314
Alcoholism	33,593	25,488	8,647	14,450	3,630	416	1,578	3.6 (3.4, 3.8)	2,754
Chronic liver disease	154,804	154,616	73,930	171,108	66,484	9,921	1,015	2.3 (2.2, 2.5)	1,419
Smokers	38,316	22,141	7,257	10,053	2,343	166	1,133	2.6 (2.4, 2.7)	2,529
Down's syndrome	2,926	451	86	32	18	4	2,016	4.6 (4.4, 4.8)	7,987
Neuromuscular/seizure disorder	49,368	18,445	6,955	17,704	9,684	2,391	1,669	3.8 (3.6, 4.0)	2,483
Short gestation/low birthweight	1,607	0	4	0	0	0	622	1.4 (1.3, 1.5)	0
Rheumatoid	48,367	35,563	16,113	41,657	21,240	4,309	1,164	2.6 (2.5, 2.8)	1,696
Rheumatoid arthritis	26,323	28,126	14,121	37,928	20,107	4,162	1,193	2.7 (2.6, 2.9)	1,582
Lupus	1,624	794	272	558	176	22	2,402	5.5 (5.2, 5.7)	3,777
Crohn's	21,202	7,128	1,892	3,508	1,098	134	990	2.2 (2.1, 2.4)	1,796
**No. of At-Risk Conditions**									
1	985,854	449,881	192,472	449,759	226,119	56,990	827	1.9 (1.8, 2.0)	1,174
2	181,063	148,772	80,216	217,993	126,693	32,402	1,485	3.4 (3.2, 3.5)	2,039
>= 2	211,864	198,396	112,146	312,062	182,233	43,853	1,665	3.8 (3.6, 4.0)	2,414
>= 3	30,801	49,624	31,930	94,070	55,541	11,451	2,721	6.2 (5.9, 6.4)	3,539
**High-Risk**	181,558	132,436	81,593	289,418	204,649	55,128	1,411	3.2 (3.0, 3.4)	2,559
Cochlear implant	719	542	332	1,156	926	373	1,669	3.8 (3.6, 4.0)	2,768
Functional/anatomic asplenia	11,350	3,580	1,721	4,290	2,122	450	1,665	3.8 (3.6, 4.0)	4,134
HIV	7,940	2,692	1,194	3,519	2,227	446	1,952	4.4 (4.2, 4.6)	2,192
Chronic renal failure	38,170	36,522	24,108	104,679	103,639	34,528	1,776	4.0 (3.8, 4.2)	3,313
Immunosuppressants	84,727	81,587	54,650	188,504	113,412	24,503	1,779	4.0 (3.9, 4.2)	2,787
Malignant neoplasms	82,755	80,199	54,163	187,200	113,247	24,477	1,556	3.5 (3.4, 3.7)	2,557
Solid organ transplantation	2,574	1,850	802	1,859	238	26	8,509	19.3 (18.9, 19.7)	12,056
Congenital immunodeficiency	38,927	10,800	3,505	8,127	3,835	712	1,123	2.5 (2.4, 2.7)	2,935
Diseases of white blood cells	9,468	5,779	2,640	7,150	3,305	640	1,986	4.5 (4.3, 4.7)	3,063

*Relative to healthy counterparts
